# Basal ganglia correlates of fatigue in young adults

**DOI:** 10.1038/srep21386

**Published:** 2016-02-19

**Authors:** Seishu Nakagawa, Hikaru Takeuchi, Yasuyuki Taki, Rui Nouchi, Yuka Kotozaki, Takamitsu Shinada, Tsukasa Maruyama, Atsushi Sekiguchi, Kunio Iizuka, Ryoichi Yokoyama, Yuki Yamamoto, Sugiko Hanawa, Tsuyoshi Araki, Carlos Makoto Miyauchi, Daniele Magistro, Kohei Sakaki, Hyeonjeong Jeong, Yukako Sasaki, Ryuta Kawashima

**Affiliations:** 1 Department of Psychiatry, Tohoku Pharmaceutical University, Sendai, Japan; 2 Department of Functional Brain Imaging, Institute of Development, Ageing and Cancer, Tohoku University, Sendai, Japan; 3 Division of Developmental Cognitive Neuroscience, Institute of Development, Ageing and Cancer, Tohoku University, Sendai, Japan; 4 Division of Medical Neuroimaging Analysis, Department of Community Medical Supports, Tohoku Medical Megabank Organization, Tohoku University, Sendai, Japan; 5 Department of Nuclear Medicine and Radiology, Institute of Development, Ageing and Cancer, Tohoku University, Sendai, Japan; 6 Human and Social Response Research Division, International Research Institute of Disaster Science, Tohoku University, Sendai, Japan; 7 Smart Ageing International Research Center, Institute of Development, Ageing and Cancer, Tohoku University, Sendai, Japan; 8 Department of Adult Mental Health, National Institute of Mental Health, National Center of Neurology and Psychiatry, Kodaira, Tokyo, Japan; 9 Department of Psychiatry, Tohoku University Graduate School of Medicine, Sendai, Japan; 10 Japan Society for the Promotion of Science, Tokyo, Japan; 11 Graduate School of Arts and Sciences, The University of Tokyo, Tokyo, Japan

## Abstract

Although the prevalence of chronic fatigue is approximately 20% in healthy individuals, there are no studies of brain structure that elucidate the neural correlates of fatigue outside of clinical subjects. We hypothesized that fatigue without evidence of disease might be related to changes in the basal ganglia and prefrontal cortex and be implicated in fatigue with disease. We aimed to identify the white matter structures of fatigue in young subjects without disease using magnetic resonance imaging (MRI). Healthy young adults (n = 883; 489 males and 394 females) were recruited. As expected, the degrees of fatigue and motivation were associated with larger mean diffusivity (MD) in the right putamen, pallidus and caudate. Furthermore, the degree of physical activity was associated with a larger MD only in the right putamen. Accordingly, motivation was the best candidate for widespread basal ganglia, whereas physical activity might be the best candidate for the putamen. A plausible mechanism of fatigue may involve abnormal function of the motor system, as well as areas of the dopaminergic system in the basal ganglia that are associated with motivation and reward.

The prevalence of chronic fatigue is approximately 20% in some developed countries^1^ and more than 33% in Japan^2^. Chronic fatigue is sometimes irreversible, and the compensation mechanisms that are useful in reducing acute fatigue are not effective^3^. Chronic fatigue has been associated with an increase in traffic accidents due to inattention, and contributes to mental health issues, such as depression, burnout syndrome^4^, and *karoshoi* (death due to overworking)^5^. A younger age was related to fatigue levels in non-clinical samples that included Australian patients aged 18 to 70 years^6^. A high prevalence of fatigue was also demonstrated among graduate students in Taiwan (46.6%)^7^. Fatigue can have numerous implications on an individual’s health and well-being; thus, it is important to elucidate the mechanisms of fatigue in young adults.

Fatigue may be defined as the failure to initiate and sustain attention-oriented tasks and physical activities requiring self-motivation^8^. A loss of motivational influence from striato–thalamic inputs to the frontal lobe is integral to the development of fatigue^8^,^9^. Moreover, the evaluation of predicted rewards and energy costs might be central to the phenomenon of mental fatigue^9^. When tasks must be performed for a prolonged period, the amount of energy that must be invested in performing the task increases compared to the potential rewards, resulting in a decrease in motivation^9^. Dopamine is involved in the control of motivational processes^10^ and reward-seeking behaviour^11^. Thus, a disruption within the dopamine system has been proposed as a common mechanism underlying fatigue^12^.

Previous functional imaging studies of fatigue with healthy individuals have predominantly addressed the relationship between certain brain regions and the subjective feeling of acute fatigue^13^,^14^,^15^,^16^. In those studies, neural activity during attention-demanding tasks decreased in the ventrolateral prefrontal cortex (PFC)^14^ and the posterior parietal cortex^15^ but increased in the cerebellar, temporal, cingulate and frontal regions^15^ and in the medial orbitofrontal cortex^16^. Functional imaging studies in patients with chronic fatigue syndrome and multiple sclerosis have suggested the basal ganglia and frontal lobes play a role in chronic fatigue^17^. As far as we know, all prior studies of brain structure and fatigue are clinical studies that examined patients with chronic fatigue syndrome^18^,^19^, multiple sclerosis^20^,^21^, Parkinson’s disease^22^ or fibromyalgia^23^. These studies identified a direct relationship between brain structure and the degree of fatigue. It was demonstrated that the degree of fatigue showed a relationship with a decline in total grey matter volume linked to a reduction in physical activity (an element of fatigue)^18^, white matter decreases in the midbrain^19^, decreases in tissue perfusion in the deep grey matter^20^, cortical atrophy of the parietal lobe^21^, and lower grey matter density in the left supplementary motor area^23^. However, no significant correlation was identified between brain structure and the severity of apathy (a symptom similar to fatigue) in patients with Parkinson’s disease^22^. Interestingly, the combination of multimodal magnetic resonance imaging (MRI), such as mean diffusivity (MD) and fractional anisotropy (FA), has been shown to be the best discriminator between patients with Parkinson’s disease and healthy controls^24^.

Although fatigue is common during adolescence^25^, there are few studies that have examined fatigue in younger populations^8^,^26^. However, to our knowledge, there are no studies that have attempted to identify the anatomical correlates of fatigue in individuals outside of clinical subjects. Structural imaging studies are suitable measures for investigating the neural correlates of fatigue because the results of such studies are not limited to the regions engaged in a specified task.

In this study, we used voxel-based morphometry (VBM) to assess regional grey matter density (rGMD) and regional white matter density (rWMD) to identify the neural correlates of fatigue. We also used voxel-based FA for diffusion tensor imaging (DTI)^27^ to assess whether WM structural integrity is associated with fatigue. FA is interpreted as an indicator of WM pathway strength or integrity^27^. In addition, we used MD to examine white matter in healthy subjects to determine the neural correlates of fatigue. There are three diffusivities, i.e., the diffusion coefficient along the direction of maximal diffusion (axial diffusivity; λ1) and the diffusion coefficients along two orthogonal directions embedded in the plane perpendicular to the main diffusion direction (λ2 and λ3)^28^. The average diffusivity of λ1, λ2 and λ3, known as MD, can be inferred from the overall dimensions of the diffusion ellipsoid^28^. MD, which is another measure of DTI, is the rate of diffusivity and a direction-independent measure of the average diffusivity reflecting water motility in a voxel. Reduction of MD is considered to reflect tissue changes caused by neural plasticity, which include astrocyte swelling, synaptic changes, dendritic spine changes, and angiogenesis^29^,^30^. Accordingly, we hypothesized that the degree of fatigue without disease may be related to basal ganglia and prefrontal cortex (PFC) function, and this relationship can be demonstrated using rGMD, rWMD, FA and especially MD in clinical cases with fatigue^8^. The purpose of this study was to identify the anatomical correlates of fatigue in young people without mental or physical disease.

## Results

### Behavioural data

**Figure 1:**
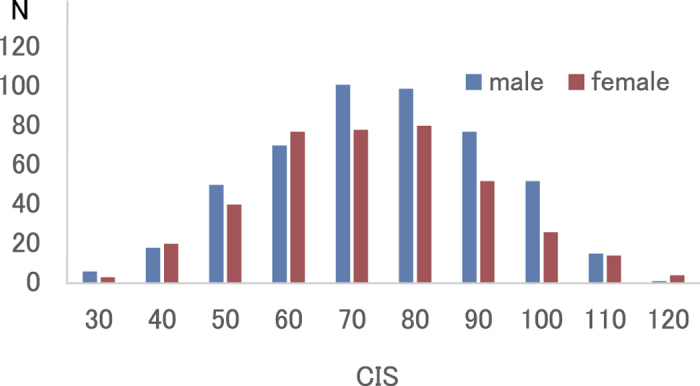
Distribution of CIS scores for men and women (n = 883). Histograms show the distributions of CIS scores for men and women. Abbreviations: CIS, Checklist Individual Strength Questionnaire.

Table 1 shows the mean and standard deviation (SD) for age, Raven’s advanced progressive matrix scores and Checklist Individual Strength Questionnaire (CIS) scores for study participants. Figure 1 shows the distributions of CIS scores in men and women. Between men and women, there was a significant difference in Raven’s Advanced Progressive Matrix (RAPM) scores (*p* < 0.05, one-way analysis of variance [ANOVA]), but not in CIS scores (*p* = 0.089). As the data in presented in Table 2 indicate, the scores for all four elements of fatigue (subjective feeling of fatigue, concentration, motivation and physical activity) were significantly and positively correlated to one another (**p** < 0.05, two-tailed corrected using the Bonferroni method).

### MRI data

#### Analysis of VBM

We found no significant correlations between CIS scores and rGMD or rWMD.

#### Analysis of FA

We found no significant correlations between CIS scores and FA.

#### Analysis of MD

**Figure 2:**
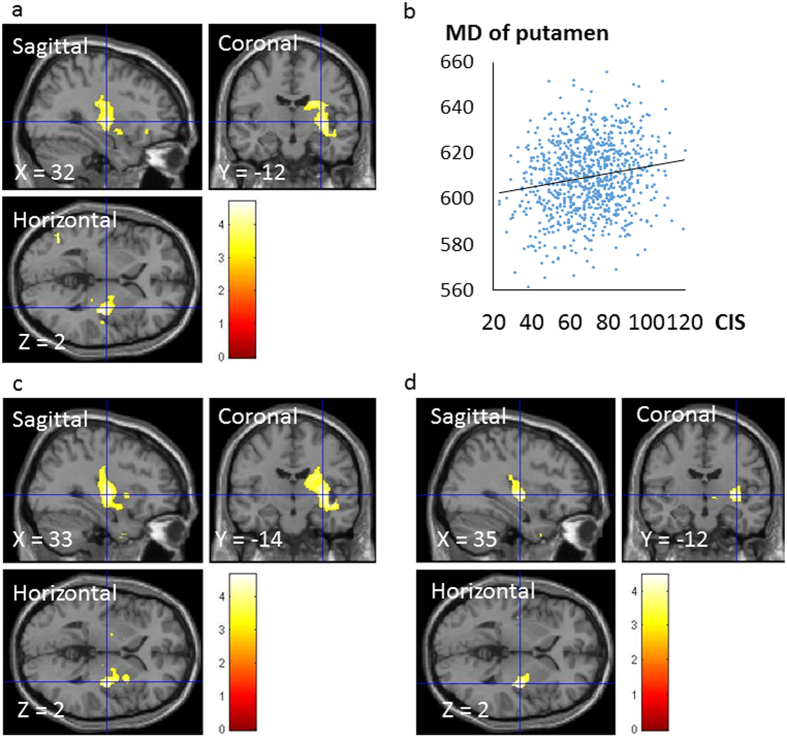
Regions showing a correlation between MD and scores on CIS, motivation and physical activity subscores. The red-to-yellow colour scale indicates the *t*-score for the positive correlation between MD and CIS scores (*p* < 0.001, uncorrected). Regions showing correlations were overlaid on a single T1 image in the SPM5 toolbox. Areas of significant correlations are shown in the right putamen (**a**). Scatter plots illustrating the relationship between mean MD and CIS scores (**b**). A cluster with significant correlations was seen in the right putamen and spanned from the palladium to the caudate. We set the statistical significance of analyses at *p* < 0.05, which was corrected for multiple comparisons at the adjusted cluster level with an underlying voxel level of *p < *0.001, uncorrected. The areas of the significant correlations are shown in the right putamen when the red-to-yellow colour scale indicates the *t*-score for the positive correlation between MD and the motivation subscore (*p* < 0.001, uncorrected) (**c**). The areas of significant correlations are shown in the right putamen when the red-to-yellow colour scale indicates the *t*-score for the positive correlation between MD and physical activity subscore (*p* < 0.001, uncorrected) (**d**). Abbreviations: CIS, Checklist Individual Strength Questionnaire; MD, mean diffusivity.

A whole-brain multiple regression analysis that controlled for sex, age, RAPM and both rGMD and regional cerebrospinal fluid density (rCSFD) at each voxel revealed a significant positive correlation between CIS scores and MD at areas corresponding to the right putamen that spanned from the palladium to the caudate (x, y, z = 32, −12, 2; *t* = 4.69; *p* < 0.001, k = 2449; corrected for multiple comparisons at the cluster with a cluster-determining threshold of *p* < 0.001, uncorrected) (Fig. 2a,b). Significant positive correlations were detected between the motivation subscores in areas corresponding to the right putamen that spanned from the palladium to the caudate (x, y, z = 33, −14, 2; *t* = 4.68; *p* < 0.001, k = 3503; corrected for multiple comparisons at the cluster with a cluster-determining threshold of *p* < 0.001, uncorrected) (Fig. 2c). Significant positive correlations were also observed between the physical activity subscores in areas corresponding to the right putamen (x, y, z = 35, −12, 2; *t* = 4.47; *p* < 0.001, k = 651; corrected for multiple comparisons at the cluster with a cluster-determining threshold of *p* < 0.001, uncorrected) (Fig. 2d).

## Discussion

To our knowledge, this study is the first to investigate an association between fatigue and brain structures in healthy individuals at the whole-brain level. Consistent with our hypothesis, we found that fatigue scores were associated with larger MD values (but not for rGMD or rWMD) in the basal ganglia, which included the putamen, pallidus and body of the caudate. That is, the degree of fatigue without disease was associated with changes in the basal ganglia, thus implicating an altered brain structure as a cause of fatigue in clinical cases. Furthermore, motivation was the best candidate for widespread basal ganglia, whereas physical activity might be the best candidate for the putamen.

First, we should discuss the mechanism through which the basal ganglia, including the putamen, pallidus and body of the caudate, are closely related to fatigue. As explained in the introduction, motivation and reward are related to fatigue^8^,^12^,^31^. Humans and animals will quickly take action when they expect the action will lead to a reward, and this action reflects their motivation^10^. In the basal ganglia, the percentage of tonically active neurons that respond to an action is higher in the putamen than in the caudate nucleus, especially in anticipation of a reward^32^. The putamen has been implicated primarily in motor control and learning habits and skills^33^,^34^. Activation of the ventral pallidum can lead to reward and enhanced motivation via phasic bursts of excitation in response to an incentive or hedonic stimuli^35^. The vigour scale of the Profile of Mood States (POMS) appears to be the most widely used and accepted measure of the energy mood state, and it is also a valid measure for nutrition-related research, such as studies of caffeine intake^36^. Among the mood states included in the POMS, only factors related to vigour were negatively correlated with MD in widespread regions that included the putamen, pallidus and body of the caudate^37^. Hence, insufficient functioning of the putamen and ventral pallidum leads to a loss of motivation and physical activity.

Second, our aforementioned findings show two possibilities (i.e., cause and effect). One finding is that a naturally higher MD (lower neuronal density) in the basal ganglia reflects a dysfunction of the basal ganglia that might cause fatigue (cause). Furthermore, the putamen, ventral pallidum and body of the caudate are associated with a loss of reward and motivation and are therefore related to fatigue. If these automatic functions are disrupted, then additional energy might be required to execute complex motor programmes, and a subsequent loss of motivation could occur. Hence, dysfunctions of the basal ganglia might lead to fatigue. This idea is consistent with the theory that fatigue results from a failure to integrate limbic inputs and motor functions in the basal ganglia, which subsequently affects the striatal–thalamic–frontal cortical system^8^. The other possibility is that fatigue affects the structure of the putamen (effect); fatigue may increase MD (decreasing neuronal density) in the basal ganglia. This idea is consistent with the fact that prolonged stress produces opposing effects on structural plasticity, notably the growth of dendrites and spines in the amygdala^38^, because repetitive and prolonged stress seems to cause fatigue^39^.

We should explain the mechanism through which MD alone could detect the neural correlates of fatigue. In the basal ganglia, MD showed a positive relationship with fatigue. From the molecular point of view, an increased MD, which is an increased water diffusivity measured on MRI, is related to a decreased tortuosity and increased volume fraction of the fast diffusivity extracellular compartment. Interestingly, the combination of MD, FA and R2* (inverse of relaxation times, i.e., relaxation rates values) in the dopaminergic system has been shown to be the best discriminator between patients with Parkinson’s disease and healthy controls^24^. There was a significant negative correlation between dopamine synthesis capacity and MD in the posterior caudate and putamen using MD with positron emission tomography (PET)^40^. Assuming that MD reflects the density of widespread axonal terminals in the striatum, dopamine synthesis may be related to the density of dopaminergic neuronal fibres^40^. Hence, MD could detect neural plasticity, especially in the dopaminergic system. Further, we could speculate the neural mechanism of fatigue based on two important aspects of fatigue, i.e., motivation and reward^8^,^9^. When a reward is greater than expected, the firing rates of certain dopaminergic neurons increase, which consequently increases motivation for the reward^11^. Interestingly, Dobryakova *et al.* reported that dopamine may have an important role in fatigue and suggested that fatigue results from disruption of communication between the striatum and PFC^41^. Thus, MD seems to be more sensitive for identifying the neural correlates of fatigue than rGMD, rWMD or FA because the relationships among fatigue, motivation and reward are based on the dopaminergic system. However, in present report, we can only speculate on the relationships among fatigue, motivation, reward and dopamine functioning in the basal ganglia because dopamine was not measured in the present study. Future studies involving more direct measures of dopamine functioning, such as PET, should examine these relationships.

Finally, there are a few limitations of this study that should be mentioned. Because the present study used a cross-sectional design, the results cannot be used to determine the causality between fatigue and the basal ganglia. Thus, to overcome this limitation, a prospective study that confirms such causality is necessary. Furthermore, we used young healthy subjects who possessed high levels of education, and such individuals might be more likely to demonstrate a high degree of plasticity.

In conclusion, fatigue without disease might result from changes in the basal ganglia, which therefore implicates the basal ganglia in fatigue in clinical cases. A plausible mechanism of fatigue may involve motivation and physical activity for maintaining performance. The neural correlates of fatigue in non-clinical and clinical subjects might overlap.

## Methods

### Subjects

Eight hundred and eighty-three healthy, right-handed individuals (489 males and 394 females) participated in this study. The present study was a part of our ongoing project to investigate associations among brain imaging data, cognitive function, aging, genetics and daily habits. The mean age of the subjects was 20.7 years (SD, 1.81). All of the subjects in our study were university or post-graduate students with normal vision, no history of neurological or psychiatric illness, and no report of recent psychoactive or antipsychotic drug use. Handedness was evaluated using the Edinburgh Handedness Inventory^42^. Written informed consent was obtained from each subject for the projects in which they participated. The procedures for all studies were approved by the Ethics Committee of Tohoku University. All experiments were performed in accordance with the approved guidelines. For more details regarding the study procedures, see the Supplemental Methods.

### Psychological outcome measures

#### Fatigue assessment

The CIS, which was developed by Vercoulen *et al.*^43^, is the most frequently used fatigue questionnaire worldwide^43^,^44^. Further, the questionnaire has been used in patients other than those who suffer from chronic fatigue syndrome^45^,^46^ and in healthy populations that included graduate students^26^ and working individuals^44^,^47^. The CIS is divided into four dimensions: subjective feeling of fatigue, motivation, activity and concentration. The CIS consists of 20 statements. Examples of these statements are as follows: “I feel tired”, “I do quite a lot within a day”, “I feel very active” and “I can concentrate well”. The total score for the CIS is an index of fatigue^44^,^47^, with a higher score indicating a higher degree of fatigue. In the present study, participants were asked to rate any subjective symptoms that they perceived themselves as having during the previous 2 weeks using a rating scale that ranged from 1 to 7. Participants were administered the Japanese version of the CIS, which was translated into Japanese by Aratake *et al.*^47^. Using a cut-off score of 76, as suggested by a previous study^48^, 317 participants (35.9%; men: 190, 38.9%; women: 127, 32.2%) were regarded as possible chronic fatigue. For more details regarding the study procedures, see the Supplemental Methods.

### Assessment of psychometric measures of general intelligence

The RAPM, which is the best measure of general intelligence^49^, was used and adjusted to examine the effect of general intelligence on brain structures^50^,^51^,^52^,^53^,^54^. This measure was also used to exclude the possibility that a significant correlation between MD and CIS scores was caused by (a) an association between CIS scores and general intelligence or (b) an association between MD and general intelligence.

### Behavioural data analyses

Behavioural data were analysed with the IBM SPSS Statistics 22.0 software package (IBM Corp.; Armonk, NY, USA). Differences between men and women in age and the scores for cognitive measures (RAPM and CIS) were analysed with one-way ANOVA. A two-tailed *p* value <0.05 was considered to indicate statistical significance. We also used Pearson’s correlation coefficient to test for correlations among feelings of fatigue, concentration, motivation, physical activity scores and MD in the significant cluster in relation to the CIS scores. A two-tailed *p* value <0.05 that was corrected using the Bonferroni method was deemed statistically significant.

### Image acquisition

MRI data were acquired using a 3T Philips Achieva scanner.

### Scan for VBM

Three-dimensional, high-resolution, T1-weighted images (T1WI) were collected using a magnetisation-prepared rapid gradient-echo (MPRAGE) sequence. The parameters were as follows: 240 × 240 matrix, TR = 6.5 ms, TE = 3 ms, TI = 711 ms, FOV = 24 cm, 162 slices, in plane resolution = 1.0 × 1.0 mm, slice thickness = 1.0 mm and scan duration of 483 s.

### Scan for FA and MD

Diffusion-weighted data were acquired using a spin-echo EPI sequence (TR = 10293 ms, TE = 55 ms, FOV = 22.4 cm, 2 × 2 × 2 mm^3^ voxels, 60 slices, SENSE reduction factor = 2, number of acquisitions =1). The diffusion weighting was isotropically distributed along 32 directions (*b* value = 1,000 s/mm^2^). For more details regarding these procedures, see the Supplemental Methods.

### Pre-processing and analysis of structural data

#### VBM

Pre-processing of the MRI data was performed using Statistical Parametric Mapping software (SPM12; Wellcome Department of Cognitive Neurology, London, UK) and following the protocol described for VBM analysis in our previous report^55^. For more details regarding these procedures, see the Supplemental Methods.

### FA and MD

Pre-processing and analysis of imaging data were performed using SPM8 implemented in MATLAB. FA and MD maps were calculated from the collected images using a commercially available diffusion tensor analysis package on the MR consol. These procedures involved correction for motion and distortion caused by eddy currents^56^. Calculations were performed according to a previously described method^27^. For more details regarding these procedures, see the Supplemental Methods.

### Statistical group-level analysis of imaging and behavioural data

The whole-brain multiple regression analysis assessed the association between rGMD and CIS scores using SPM12. The whole-brain multiple regression analysis was performed using SPM12 and assessed the relationship between rWMD and CIS scores. The whole-brain multiple regression analysis was performed using SPM8 and assessed the relationship between FA and CIS scores. The covariates included sex, age, RAPM scores and total intra-cranial brain volume (TIV: total GM volume + total WM volume + total cerebrospinal fluid volume). For each covariate, the “overall mean” was used for mean centring.

For analyses involving MD, we used the biological parametric mapping (BPM) toolbox^57^, which is an extension software of SPM5, the latest available version for the BPM toolbox (Wellcome Department of Cognitive Neurology, London). Using the BPM toolbox, we performed multimodality voxel-wise multiple regression analyses adjusted for the effects of rGMD and rCSFD to investigate associations between MD and CIS scores. These values were adjusted to exclude the possibility that the extent of GM, WM or CSF itself affected the results rather than MD (in the areas analysed, tissues were either GM, WM or CSF; thus, regressing out the effects of the rGMD or rCSFD should address these issues). The BPM toolbox can perform multiple regression analyses using multimodal images. We performed a voxel-by-voxel regression analysis, and in this analysis, the dependent variable at each voxel was the MD value at that voxel. The independent variables included the rGMD value and the rCSFD map at that voxel, as well as age, sex, RAPM score and the CIS score. The analyses were limited to areas within the grey + white matter mask that was created using the procedures described above. Three of the Pearson’s correlation coefficients among the four subscales were >0.5. Accordingly, multicollinearity may be doubted among the four subscales based on the multiple regression analysis, which can have severe effects on parameter estimates. However, we performed voxel-by-voxel regression analyses using the same covariates and added the four CIS subscales simultaneously, rather than the total CIS score. No significant MD was related to any of the CIS subscores. Furthermore, we also performed four voxel-by-voxel regression analyses using the same covariates mentioned above and added each CIS subscore in turn, rather than the total CIS score, to determine which subscore was the best candidate for the region. We set the statistical significance for these analyses at *p* < 0.05, and corrected for multiple comparisons at the adjusted cluster level with an underlying voxel level of *p < *0.001, uncorrected.

## Additional Information

**How to cite this article**: Nakagawa, S. *et al.* Basal ganglia correlates of fatigue in young adults. *Sci. Rep.* **6**, 21386; doi: 10.1038/srep21386 (2016).

## Supplementary Material

Supplementary Information

## Figures and Tables

**Table 1:** Sex differences in age and scores on the RAPM and CIS; and one-way ANOVA results.

Measure	Male		Female	
	Mean	SD	Mean	SD
Age	20.79	1.94	20.56	1.63
RAPM	28.89	3.66	28.06	3.78
CIS	70.37	17.52	68.34	11.77

Abbreviations: CIS, the Checklist Individual Strength Questionnaire; RAPM, Raven’s Advanced Progressive Matrix; SD, standard deviation.

**Table 2:** Pearson’s correlation coefficients among the four fatigue elements.

	Feeling	Concentration	Motivation	Physical activity	VIF
Feeling	–				1.204
Concentration	0.391^*^	–			1.654
Motivation	0.273^*^	0.445^*^	–		1.498
Physical activity	0.310^*^	0.573^*^	0.551^*^	–	1.786

Abbreviations: feeling, subjective feeling of fatigue; VIF, variance inflation factor.

*p < 0.001 (two-tailed correction using the Bonferroni method).
