# Supplementary material for: Basal ganglia correlates of fatigue in young adults
Source: Sci Rep. 2016 Feb 19;6:21386. doi: 10.1038/srep21386 (PMC4759547; doi:10.1038/srep21386)
Supplement: Supplementary Information [file srep21386-s1.doc]

**Basal ganglia correlates of fatigue in young adults**

Seishu Nakagawa^1,2^, Hikaru Takeuchi^3^, Yasuyuki Taki^3,4,5^, Rui Nouchi^6,7^, Yuka Kotozaki^7^, Takamitsu Shinada^2^, Tsukasa Maruyama^2^, Atsushi Sekiguchi^2,4,7,8^, Kunio Iizuka^2,9^, Ryoichi Yokoyama^2,10^, Yuki Yamamoto^2^, Sugiko Hanawa^2^, Tsuyoshi Araki^7^, Carlos Makoto Miyauchi^2,11^, Daniele Magistro^2,10^, Kohei Sakaki^2^, Hyeonjeong Jeong^2,10^, Yukako Sasaki^2^, Ryuta Kawashima^2,3,7^

**Institutions:**

*^1^Department of Psychiatry, Tohoku Pharmaceutical University, Sendai, Japan*

*^2^Department of Functional Brain Imaging, Institute of Development, Ageing and Cancer, Tohoku University, Sendai, Japan*

*^3^Division of Developmental Cognitive Neuroscience, Institute of Development, Ageing and Cancer, Tohoku University, Sendai, Japan*

*^4^Division of Medical Neuroimaging Analysis, Department of Community Medical Supports, Tohoku Medical Megabank Organization, Tohoku University, Sendai, Japan*

*^5^Department of Nuclear Medicine and Radiology, Institute of Development, Ageing and Cancer, Tohoku University, Sendai, Japan*

*^6^Human and Social Response Research Division, International Research Institute of Disaster Science, Tohoku University, Sendai, Japan*

*^7^Smart Ageing International Research Center, Institute of Development, Ageing and Cancer, Tohoku University, Sendai, Japan*

*^8^Department of Adult Mental Health, National Institute of Mental Health, National Center of Neurology and Psychiatry, Kodaira, Tokyo, Japan.*

*^9^Department of Psychiatry, Tohoku University Graduate School of Medicine, Sendai, Japan*

*^10^Japan Society for the Promotion of Science, Tokyo, Japan*

*^11^Graduate School of Arts and Sciences, The University of Tokyo, Tokyo, Japan.*

**Supplemental Methods**

**Subjects**

Data from subjects who participated in this study were included in other studies unrelated to the focus of this study ^1^. Some of the subjects who participated in this study were also subjects in interventional studies (psychological data and imaging data were recorded before the intervention was used in this study) ^2^. Psychological tests and MRI scans not described in this study were performed with the tests described for this study (psychological data and imaging data recorded before the intervention were used in this study). The subjects were recruited through advertisements on bulletin boards at Tohoku University or e-mails to participants of previous experiments in our laboratory. All university students underwent a health examination that included an eyesight exam. In addition, we tested eyesight using an auto refractometer (Shin-Nippon ACCUREF 8001 Auto Refractometer, Ajinomoto Trading Inc., Tokyo). At the time of recruitment, we informed potential participants that individuals with certain diseases could not participate in the experiment, and they were continuously notified of such exclusion criteria. Thus, subjects meeting the exclusion criteria should have been excluded before they came to the lab; however, subjects meeting the exclusion criteria were removed from the study. The number of subjects excluded during the recruitment processes could not be verified, because in many cases where the subject dropped out of the study, the reason for dropout was unknown, and there was no record for the informal preliminary contacts.

**Fatigue assessment**

A confirmatory factor analysis was carried out to determine whether the factor structure of the CIS was similar to that of the original CIS, which Vercoule et al. developed ^3^. Cronbach’s alpha for the total CIS score was high (0.91). When the subjects had completed the CIS on two occasions, with an interval of 3 days, the test-retest reliability assessed with an intra-class correlation coefficient was significantly high (0.82) ^4^. To validate the CIS, Aratake et al. analysed the correlation coefficients between the total CIS score and the Maslach burnout inventory-general survey exhaustion score, the Beck Depression Inventory-II score and the visual analogue scale for subjective fatigue score, which were 0.58 (*p* < 0.01), 0.66 (*p* < 0.01) and 0.63 (*p* < 0.01), respectively ^4^.

**Image acquisition**

Scan for FA and MD

Three images with no diffusion weighting (*b* value = 0 s/mm^2^) (b = 0 images) were acquired, using a spin-echo EPI sequence (TR = 10293 ms, TE = 55 ms, FOV = 22.4 cm, 2 × 2 × 2 mm^3^ voxels, 60 slices). Acquisitions for phase correction and for signal stabilisation were performed, but they were not used as part of the reconstructed images.

**Pre-processing and analysis of structural data**

**VBM**

Regional grey matter density (rGMD) and regional white matter density (rWMD) were calculated. T1WIs of each individual were segmented into 6 tissues using the default parameter settings of a segmentation algorithm implemented in Statistical Parametric Mapping software (SPM) 12 in Matlab (Mathworks Inc., Natick, MA, USA). However, there were three exceptions: affine regularization was performed in accordance with the Asian template and sampling distance (the approximate distance between sampled points when estimating the model parameters) was 1 mm, and a thorough clean option was used to remove odd voxels from segmented images. We then used a diffeomorphic anatomical registration through exponentiated lie (DARTEL) algebraic registration process implemented in SPM12. In this process, we used DARTEL-imported images of grey and white matter tissue probability maps (TPMs) created using the abovementioned segmentation process. First, the template for the DARTEL procedures was created using imaging data from 800 subjects (400 men and 400 women) who participated in this project. The resulting images were then spatially normalized to the Montreal Neurological Institute (MNI) space to obtain images with 1.5 × 1.5 × 1.5 mm^3^ voxels. In addition, we performed a volume change correction (modulation) by modulating each voxel with the Jacobian determinants derived from the spatial normalization and allowing for the determination of regional differences in the absolute amount of brain tissue. Subsequently, all images were smoothed by convolving them with an isotropic Gaussian kernel of 12-mm full width at half maximum (FWHM). The statistical significance level was set at *p* < 0.05, corrected at the non-isotropic adjusted cluster level ^5^ with an underlying voxel level of *p* < 0.0025. In this non-isotropic cluster-size test of the random field theory, a relatively higher cluster-determining threshold combined with high smoothing values of 12 mm was shown to lead to appropriate conservativeness in real data ^6^.

**FA and MD**

Subsequently, using a previously validated two-step new segmentation algorithm for diffusion images and the previously validated DARTEL-based registration process using SPM8 ^7^, all images, including grey matter segments (regional grey matter density [rGMD] map), white matter segments (regional white matter density [rWMD] map) and cerebrospinal fluid (CSF) segments (regional CSF density [rCSFD] map) of diffusion images, were normalized. The voxel size of normalized FA images, MD images and segmented images was 1.5 × 1.5 × 1.5 mm^3^.

Next, we created average images of normalized rGMD and rWMD images from the normalized rGMD and rWMD images using a subset of the entire sample (63 subjects) ^7^. Subsequently, for the analyses of MD images from the normalized images of the (a) MD, (b) rGMD and (c) rCSFD maps, we created images where areas that were least likely to be grey or white matter in our averaged normalized rGMD and rWMD images (defined by a grey matter tissue probability + white matter tissue probability < 0.99) were removed (to exclude the strong effects of CSF on MD throughout analyses). These images were then smoothed (6-mm FWHM) and carried through to the second-level analyses of MD.

**Supplementary references**

1. Takeuchi, H.*, et al.* Regional gray matter volume of dopaminergic system associate with creativity: evidence from voxel-based morphometry. *Neuroimage* **51**, 578-585 (2010).

2. Takeuchi, H.*, et al.* Effects of Multitasking-Training on Gray Matter Structure and Resting State Neural Mechanisms. *Human Brain Mapping* **35**, 3646-3660 (2014).

3. Vercoulen, J.H.*, et al.* Dimensional assessment of chronic fatigue syndrome. *J Psychosom Res* **38**, 383-392 (1994).

4. Aratake, Y.*, et al.* Development of Japanese version of the checklist individual strength questionnaire in a working population. *J Occup Health* **49**, 453-460 (2007).

5. Hayasaka, S., Phan, K.L., Liberzon, I., Worsley, K.J. & Nichols, T.E. Nonstationary cluster-size inference with random field and permutation methods. *NeuroImage* **22**, 676-687 (2004).

6. Silver, M., Montana, G. & Nichols, T.E. False positives in neuroimaging genetics using voxel-based morphometry data. *NeuroImage* **54**, 992-1000 (2011).

7. Takeuchi, H.*, et al.* White matter structures associated with empathizing and systemizing in young adults. *Neuroimage* **77**, 222-236 (2013).
